# Polyvalent Proteins, a Pervasive Theme in the Intergenomic Biological Conflicts of Bacteriophages and Conjugative Elements

**DOI:** 10.1128/JB.00245-17

**Published:** 2017-07-11

**Authors:** Lakshminarayan M. Iyer, A. Maxwell Burroughs, Swadha Anand, Robson F. de Souza, L. Aravind

**Affiliations:** a National Center for Biotechnology Information, National Library of Medicine, National Institutes of Health, Bethesda, Maryland, USA; b Microbiology Department, Biomedical Sciences Institute, University of Sao Paulo, Cidade Universitária, São Paulo, Brazil; University of Tennessee at Knoxville

**Keywords:** DNA replication, DNA-binding proteins, RNases, antirestriction, bacteriophages, biological conflicts, effectors, metallopeptidase, plasmids, transcription

## Abstract

Intense biological conflicts between prokaryotic genomes and their genomic parasites have resulted in an arms race in terms of the molecular “weaponry” deployed on both sides. Using a recursive computational approach, we uncovered a remarkable class of multidomain proteins with 2 to 15 domains in the same polypeptide deployed by viruses and plasmids in such conflicts. Domain architectures and genomic contexts indicate that they are part of a widespread conflict strategy involving proteins injected into the host cell along with parasite DNA during the earliest phase of infection. Their unique feature is the combination of domains with highly disparate biochemical activities in the same polypeptide; accordingly, we term them polyvalent proteins. Of the 131 domains in polyvalent proteins, a large fraction are enzymatic domains predicted to modify proteins, target nucleic acids, alter nucleotide signaling/metabolism, and attack peptidoglycan or cytoskeletal components. They further contain nucleic acid-binding domains, virion structural domains, and 40 novel uncharacterized domains. Analysis of their architectural network reveals both pervasive common themes and specialized strategies for conjugative elements and plasmids or (pro)phages. The themes include likely processing of multidomain polypeptides by zincin-like metallopeptidases and mechanisms to counter restriction or CRISPR/Cas systems and jump-start transcription or replication. DNA-binding domains acquired by eukaryotes from such systems have been reused in XPC/RAD4-dependent DNA repair and mitochondrial genome replication in kinetoplastids. Characterization of the novel domains discovered here, such as RNases and peptidases, are likely to aid in the development of new reagents and elucidation of the spread of antibiotic resistance.

**IMPORTANCE** This is the first report of the widespread presence of large proteins, termed polyvalent proteins, predicted to be transmitted by genomic parasites such as conjugative elements, plasmids, and phages during the initial phase of infection along with their DNA. They are typified by the presence of multiple domains with disparate activities combined in the same protein. While some of these domains are predicted to assist the invasive element in replication, transcription, or protection of their DNA, several are likely to target various host defense systems or modify the host to favor the parasite's life cycle. Notably, DNA-binding domains from these systems have been transferred to eukaryotes, where they have been incorporated into DNA repair and mitochondrial genome replication systems.

## INTRODUCTION

Cellular genomes are pitted in multilevel conflicts involving a diverse array of nonself genomes, including bacteriophages, plasmids, and conjugative transposons (1, 2). At one level of conflict, these elements utilize cellular resources to further their own replication, thereby reducing the fitness of the host genome. At another level, they might encode determinants that enhance host fitness against rival invasive elements or competing organisms. Such conflicts are particularly widespread in prokaryotes, where the DNA, unlike that in eukaryotes, is not sequestered in a separate organelle. As a result, extensive molecular “weaponry” and repair systems, which counter damage caused by such weaponry, have evolved in both prokaryotic cellular genomes and invasive elements that exploit them. In the past 2 decades, comparative genomic analyses have been particularly successful in bringing to light such adaptations in both cellular genomes (1, 3–5) and invasive elements (2, 6–8) and point to a veritable “arms race” between the two. A key commonality in the conflict-related adaptations across these systems is the presence of a striking array of effectors (sometimes termed toxins) that, with various degrees of specificity, target the macromolecules of the competing entity, such as DNA, various RNAs, proteins from core systems, cell membranes, and cell walls. Most well-characterized effectors achieve their end result through the action of one or more catalytic domains that covalently modify or cleave the target macromolecule. However, others might achieve the same end result through a catalytic activity that generates a low-molecular-weight product or via noncovalent interactions with the target molecule (9–11).

While conflict and damage repair systems encoded by cellular genomes have been subject to intense scrutiny in several recent studies (2, 11), much less is known of mechanisms deployed by invasive nonself genomes against these host responses. Although targeting of invasive elements occurs during several distinct phases of the life (infection) cycle of the parasitic element (9), we were particularly interested in the conflict mechanisms they have evolved to survive the initial phase involving the entry of the element as single-stranded DNA (ssDNA) or double-stranded DNA (dsDNA) and the period shortly thereafter (2, 7, 8). Invasive elements are particularly vulnerable at this stage because they are typically in a single copy and might not have had the time for the synthesis of defensive components by using host resources. Moreover, an invading element is not just attacked by host defenses but might also face competition from other invasive elements that are already resident in the host (e.g., immunity conferred by lysogenic phages). These competing elements too are likely to deploy their weaponry right at the time of invasion to prevent establishment of the new element in the host (12). Consistent with the proposal that this is indeed a phase of intense biological conflict, several studies have shown that invasive elements often transfer, along with their genomic DNA, a diverse array of proteins that facilitate their survival. In conjugative transposons and mobile plasmids using the type IV secretion system (T4SS) and related secretion systems for DNA transfer, protein effectors either “escort” the DNA or are independently transferred through the T4SS (13–17). Phages package effector proteins into their capsid head and inject them along with DNA into the host cell (18–20).

Proteins identified in prior studies that are transferred during invasion can be grouped into five broad categories. (i) DNA processing and transfer proteins are seen primarily in conjugative transposons and plasmids and are intimately involved in the mechanics of conjugation. The key protein here is a relaxase with a catalytic domain of the rolling-circle replication (RCR) superfamily that nicks the element's DNA, forming a covalent linkage with a single strand of the element's DNA. The DNA is then unwound, transferred along with the linked relaxase through the T4SS, and religated by the relaxase to form a single-stranded circle in the new host (14, 15). (ii) Host-modifying enzymes have been studied primarily in phages, and the prototype is the phage T4 Alt protein, an ADP-ribosyltransferase (ART) that is packaged into the phage head and injected into the host cell along with viral DNA. Alt ADP-ribosylates the host RNA polymerase (RNAP) subunits and probably other proteins, including translation factors, metabolic enzymes, and chaperones (21), with the host RNAP consequently switching specificity to transcribe phage DNA (22, 23). Further evidence comes from the MuF domain proteins (e.g., phage Mu F [gp30] and Bacillus phage SPP1 gp7), which are structural components of the head in phages utilizing the portal terminase packaging system (24, 25). The MuF domain is fused to a number of enzymatic domains that are likely to function as effectors delivered into the host cell by phages (10, 26). (iii) Antirestriction proteins are deployed by both phages and mobile elements and target the barrier imposed on invasive DNA by restriction-modification (R-M) systems from the host or other resident elements. One mode of action, typified by the phage T7 OCR (overcome classical restriction), phage T4 IPI (internal protein I), phage lambda Ral (restriction alleviation), and ArdA and ArdB (alleviation of restriction of DNA) proteins of conjugative transposons and self-transmissible plasmids, is physical interaction with restriction enzymes to inhibit their activity (27–30). In contrast, the phage P1 DarA and DarB (defense against restriction) proteins and the plasmid ArdC proteins bind the transferred DNA, protecting it against R-M systems (31, 32). (iv) Anti-CRISPR mechanisms have been identified primarily in phages and inhibit the CRISPR/Cas immunity system by binding various proteins in the CRISPR/Cas complex (33, 34). (v) Early life cycle components directly initiate early events of the posttransfer life cycle of both phages and mobile elements. One example is the virion-packaged RNAP of coliphage N4 and related Pseudomonas phages, which transcribes early genes (35, 36). Examples from mobile elements include the Toprim domain primases, TraC1 of IncP plasmid RP4 and SogL of Inc1 plasmid R64 (37, 38), which are transferred during conjugation and prime posttransfer DNA replication in the new host.

Given our interest in biological conflicts during invasion by nonself elements, we used comparative genomic analysis to better understand the components and diversity of systems such as those described above. In the course of this analysis, we uncovered a remarkable class of proteins that are widely distributed in phages, prophages, plasmids, and conjugative transposons and characterized by the fusion in the same polypeptide of multiple protein domains with a striking diversity of biochemical activities. Accordingly, we term them polyvalent proteins. They encompass at least 131 distinct domain types, many of which we predict to mediate an array of functions needed to establish the invasive elements in host cells, promote their replication, and overcome host defenses directed against them. We propose that these polyvalent proteins represent a hitherto undescribed general strategy used by invasive genomes in the face of the ongoing arms race with their prokaryotic hosts and rival invasive elements.

## RESULTS AND DISCUSSION

### Search strategy for recovery of early-phase effectors in invasive DNA elements.

**FIG 1:**
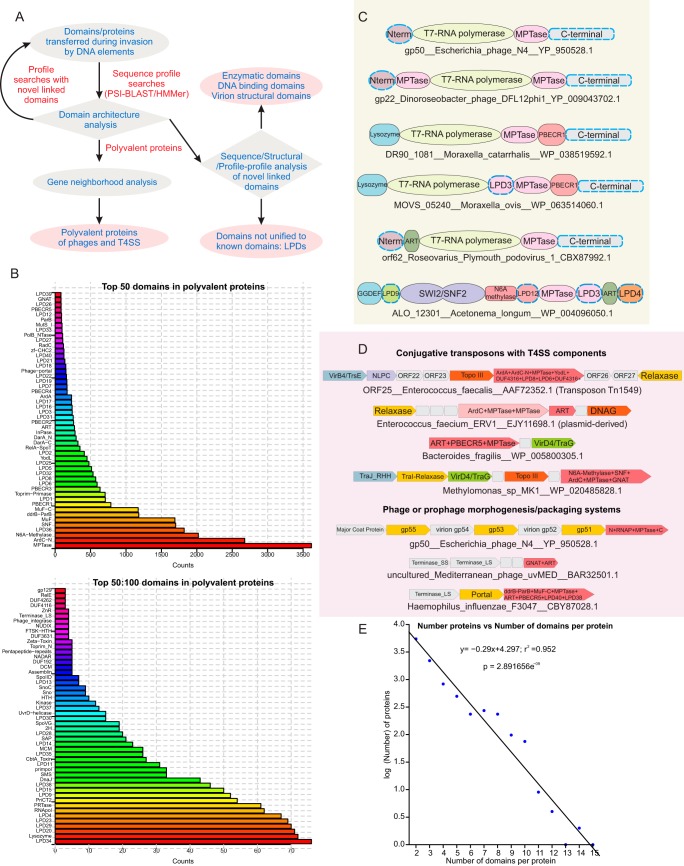
(A) Schematic showing the recursive process used to find domains in polyvalent proteins. (B) Frequency distribution of the top 100 domains observed in polyvalent proteins. (C) Examples of recovered polyvalent proteins and their domain architectures. (D) Examples of neighborhoods of polyvalent-protein-encoding genes illustrating the broad types of genome contexts. (E) Plot illustrating the number of polyvalent proteins with a given number of domains. Note that the *y* axis is on a log scale.

**FIG 2:**
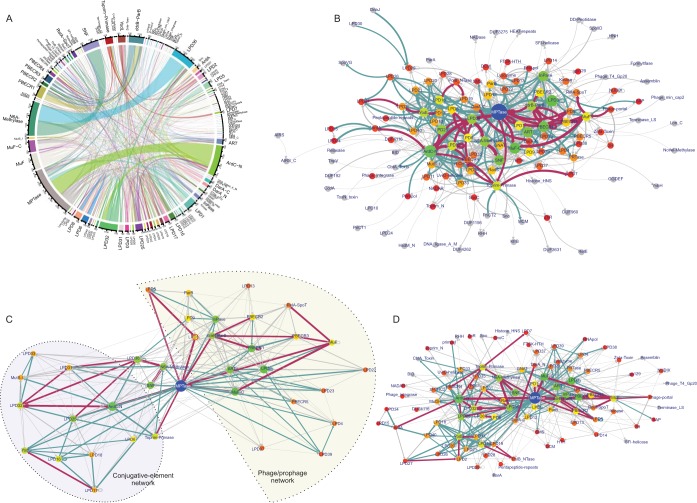
Polyvalent protein domain networks. (A) Chord diagram of domain cooccurrences in polyvalent proteins. The plot includes all cooccurrences of domains. Thus, an edge is drawn between two domains in a protein whether they are adjacent or not. (B) Domain architecture network of polyvalent proteins. Domains linked in the same protein are connected by arrows with each arrowhead pointing to the C-terminal domain. Here and in the subsequent network images, an edge is drawn only between two adjacent domains. (C) Clique subnetwork of the domain architecture network merging large cliques with seven or eight nodes. The network reveals two distinct subgraphs as described in the text. (D) Largest biconnected subnetwork of the domain architecture network. In all of the networks, the node size and color are scaled on the basis of the number of connections per node (degree). Nodes with two or fewer connections are gray. Edge thickness is based on the number of edge occurrences. Edges occurring <15 times are gray, those occurring 16 to 90 times are cadet blue, and those occurring >90 times are maroon.

To determine the distribution and diversity of proteins that are transferred during the invasion of hosts by various DNA elements (phages, plasmids, and conjugative transposons), we first initiated sequence profile searches by using a comprehensive library of previously identified exemplars of such proteins as queries (Fig. 1A). These included relaxase, phage T4 Alt, phage T7 OCR, phage T4 IPI, Ard proteins, phage P1 DarA and DarB, N4 virion RNAP, MuF, and the recently reported anti-CRISPR proteins. Preliminary searches were carried out iteratively in the nonredundant (nr) database with the PSI-BLAST and JACKHMMER programs. Several queries, such as T7 OCR, T4 IPI, and the anti-CRISPR proteins, recovered a phyletically limited set of proteins, suggesting that they probably represent lineage-specific adaptations. In contrast, searches with relaxase, phage T4 Alt, Ard proteins, phage N4 RNAP, MuF, and phage P1 DarA and DarB recovered a large, diverse array of hits. We then evaluated the gene contexts of the hits recovered to check for the presence of either (pro)phage packaging systems (late genes) or associations with the conjugative element transfer components. Thus, we distinguished proteins that are transferred during invasion from those that are either not linked to invasive elements or unlikely to be transferred with DNA. Through this analysis, we recovered a distinct and notable class of proteins with one or more of the above-mentioned domains, where they were combined in multidomain polypeptides, often of large size (>2,000 amino acids [aa]), with several other known or novel domains (hence, polyvalent proteins). Their linkage in a single polypeptide gave us contextual information, suggesting that they are likely to act together at the same time. We then comprehensively investigated the other domains found in such proteins by seeding iterative profile searches similar to the initial ones with each of the newly isolated domains. This was followed by a further detailed analysis of their gene neighborhood and domain architectural contexts. Profile-profile searches with the HHPRED program seeded with hidden Markov models generated from sequence alignments of these domains were used to recover distant homologs and determine their protein fold (Fig. 1A). Globular domains that could not be unified in any of our searches to known domains were assigned a code consisting of an LPD (large polyvalent-protein-associated domain) prefix followed by a number. By this iterative method, we assembled a comprehensive inventory of >9,928 polyvalent proteins with 131 domains (see Files S1 and S5 at ftp://ftp.ncbi.nih.gov/pub/aravind/polyvalent/polyvalent.html) whose domain architectural and gene neighborhood linkages are presented as a contextual network (Fig. 2A). The 100 most frequently occurring of these domains are found in anywhere between 3,900 and 3 distinct proteins (Fig. 1B).

**FIG 3:**
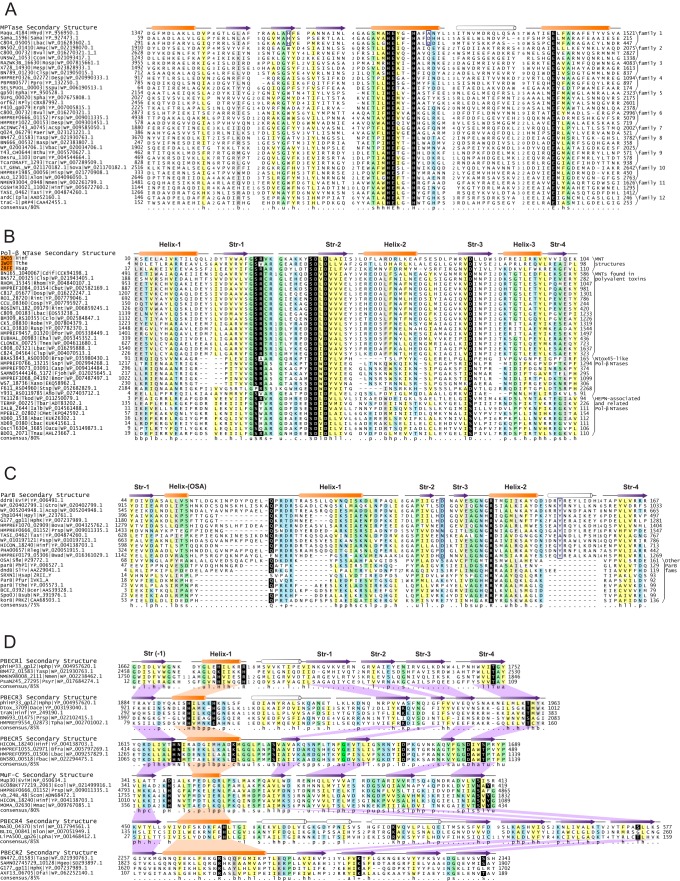
Multiple-sequence alignments of MPTase (A), Pol-β NTase (B), DdrB-ParB (C), and PBECR (D) domains. Secondary structure and element labeling is provided at the top line, and residue consensus at various percentages is provided at the bottom line. Sequences are identified on the left by gene name, organism abbreviation, and NCBI accession number separated by vertical lines and to the right by family name. Sequences of structures are labeled with PDB codes, shaded in orange. Poorly conserved secondary structure elements are colored white. Family-specific conserved residues described in the text are denoted by blue boxes. Alignments are colored as follows: h (hydrophobic), l (aliphatic), and a (aromatic) are shaded yellow; p (polar), + (positively charged), − (negatively charged), and c (charged) are shaded blue; s (small) and t (tiny) are shaded green; b (big) is shaded gray; absolutely conserved residues are in white lettering and shaded in black. For organism abbreviations, see File S4 at ftp://ftp.ncbi.nih.gov/pub/aravind/polyvalent/polyvalent.html.

We illustrate the above-described search procedure with a typical example. The large virion polymerase of coliphage N4 (gp50; accession no. YP_950528.1) is a multidomain protein with an N-terminal alpha-helical region (998 aa) involved in protein injection into the host, a middle polymerase domain that provides the core DNA-dependent RNAP activity (1,100 aa; Protein Data Bank [PDB] code 2PO4), and a C-terminal 1,400-aa region required for encapsidation (Fig. 1C) (36, 39, 40). Sequence and profile-profile searches using the N-terminal region and middle polymerase domain recovered only a T7 RNAP module that is also found in related phages and prophages. However, we found that the C-terminal region is composed of two potential domains, of which one is associated in other proteins with a greater diversity of domains. The upstream domain of this pair contains a highly conserved HEXXH motif (region, aa 2130 to 2448). Profile-profile searches with an alignment of this region unified it to the zincin-like metallopeptidase (MPTase) superfamily (e.g., the lethal factor endopeptidase; PDB code 4dv8; *P* = 4.1 × 10^−4^ in HHPRED). Domain context analysis showed that the N4-like RNAPs are always fused to one or more MPTase domains and can additionally be fused in comparable large proteins to a lysozyme/transglycosylase domain (e.g., Moraxella prophage protein DR90_1081; GenBank accession no. WP_038519592.1), a novel predicted RNase of the barnase/EndoU/colicin E5/D-RelE (BECR)-like fold (10) (see below for details; PBECR1, Moraxella prophage DR90_1081; accession no. WP_038519592.1), and a NAD^+^-dependent ART domain (Roseovarius Plymouth podovirus 1 vRNAP; accession no. CBX87992.1) (41) (Fig. 1C). The MPTase domain is also independently fused in large proteins to other globular domains such as lysozyme, ART, PBECR1 (see below), SWI2/SNF2 ATPase, DNA adenine methylase, a distinct ParB domain, and a variety of LPDs (Fig. 2; see Fig. 3A). Thus, a network of domain connections centered on the RNAP, the MPTase, and the linked domains was obtained and this was further iteratively extended as mentioned above (Fig. 1A and 2).

### Polyvalent proteins encoded by phages and mobile elements combine functionally diverse domains.

We observed that the polyvalent proteins retrieved by the above-described search procedure occur in three broad genomic contexts. (i) The first is the presence in a plasmid or conjugative transposon encoded alongside T4SS-related components (Fig. 1C). Examples include integrating conjugative elements, prototyped by the vancomycin resistance transposon Tn*1549*-like elements of Firmicutes (42) and the Tn*5253*-like elements of Streptococcus pneumoniae (43), and several conjugative plasmids from diverse prokaryotic lineages (Fig. 1D). This is the largest subset of polyvalent proteins in our collection and includes up to 40% of the proteins recovered. (ii) In the second context, up to 35% of the polyvalent proteins are encoded by phages or prophages and are likely to be packaged in the capsid (see below). These encompass a great diversity of phages, but most of them are unified by the presence of a portal terminase packaging system (26) (Fig. 1D). (iii) In the third context, the proteins, while not showing any distinguishing genome-contextual associations, show homologous domains and domain architectures syntactically identical to one of the above two types. We interpret these as being polyvalent proteins acquired by the host genome from remnants of one of the above-described invasive elements that might be deployed defensively against other invasive elements.

Polyvalent proteins contain 2 to 15 domains in a polypeptide (Fig. 1D) with the number of proteins with each additional number of domains above 2 falling exponentially (*r*^2^ = 0.95, *P* = 2.89 × 10^−9^) (Fig. 1E). Although >75% of the proteins have only two or three domains, the same domains are often found together or separately in larger polyvalent proteins with other domains, suggesting that, irrespective of their domain architectural complexity, they belong to the same functional system. Large multidomain proteins with variable domain architectures have been previously reported in multiple conflict-related contexts in prokaryotes. (i) Proteins involved in the biosynthesis of secondary metabolites (e.g., antibiotics and siderophores), especially based on peptide or polyketide skeletons, combine nonribosomal peptide synthetase or polyketide synthetase domains with several other enzymatic and nonenzymatic domains (44, 45). These domains usually act sequentially in the biosynthesis of the secondary metabolite, and the variations in the combined domains contribute to the diversity of the secondary metabolites produced by them. (ii) Secreted toxin proteins, which are some of the largest proteins in bacteria, display multidomain architectures typically with N-terminal domains related to particular secretion systems; middle domains usually composed of sequence repeats involved in packaging, presentation, or autoproteolytic processing; and C-terminal toxin domains (10, 46, 47). Most of the variability here is seen in the C-terminal toxin domains. However, the polyvalent proteins recovered in this study, while having certain domains in common with the above-described systems, did not conform to the above-described organizational themes. The domains included in these proteins broadly belong to four types, (i) known and predicted enzymatic domains with diverse activities, (ii) DNA-binding domains, (iii) domains with structural roles in phage virions, and (iv) domains for which we were unable to predict a definitive function. Strikingly, a common theme often seen in polyvalent proteins is the linking of enzymatic domains with entirely unrelated biochemical activities in the same polypeptide. A dramatic example of this is seen in a gigantic protein of prophage origin encoded by the genome of the firmicute Acetonema longum (accession no. WP_004096050.1) containing at least nine domains, of which the known enzymatic domains include the cyclic-diguanylate-generating GGDEF, SWI2/SNF2 ATPase, DNA adenine methyltransferase (MTase), zincin-like MPTase, and ART domains (Fig. 1B). This observation indicates that the domains of the polyvalent proteins are likely to target multiple macromolecules or systems, probably simultaneously, upon delivery during invasion.

We next present a systematic analysis and functional prediction (where possible) of the domains that are commonly found in polyvalent proteins, followed by an analysis of their contextual associations.

### Catalytic domains in polyvalent proteins predicted to act on other proteins.

One of the most frequently found classes of catalytic domains observed in polyvalent proteins are predicted to operate on proteins and peptides. These include peptidases and four families of protein-modifying domains, namely, the ARTs, serine/threonine/tyrosine-type protein kinases, GCN5-like acetyltransferases (GNATs), and the polymerase β (Pol-β) superfamily nucleotidyltransferases (NTases). These domains are also encountered in diverse conflict systems such as polymorphic toxin, toxin-antitoxin, antibiotic resistance, and bacterial effector systems targeting eukaryotic host proteins (10, 41, 48, 49). This favors a similar conflict-related role for their counterparts in polyvalent proteins.

### Peptidases.

Polyvalent proteins possess four distinct superfamilies of peptidases. Of these, the zincin-like MPTase is by far the most widespread and is overall the most common type of domain in polyvalent proteins. It is also the most prominent hub domain in the overall domain network of polyvalent proteins (Fig. 2A). About 36% of the polyvalent proteins in our data set have one to five MPTase domains, of which about 8% have more than one copy of the domain in the same polypeptide. A significant percentage of these proteins (52%) are associated with the ArdC-N domain (see below) and make up about 32% of the 514 distinct nonredundant domain architectural types with MPTases. The MPTase domains are found in the N- or C-terminal or middle region of the proteins, and in those with two or more MPTase domains, they are often interspersed between other domains.

The core fold of the zincin-like MPTase domain is characterized by the presence of a three-stranded β sheet flanked by an N-terminal helix and two C-terminal helices (10, 50). The first of the two conserved C-terminal helices contains the catalytic HEXXH motif, while the second contains a glutamate residue (Fig. 3A). The two histidines, the glutamate from the C-terminal helix, and a water molecule coordinate a Zn^2+^ ion in the active-site pocket, whereas the glutamate in the HEXXH motif is predicted to function as the general base that activates a water molecule for proteolysis (50). Beyond these conserved residues, the MPTases from polyvalent proteins show multiple variations in the configurations of their active site, additional conserved residues likely to contribute to the active-site pocket, and variable inserts between the two C-terminal conserved helices (Fig. 3A). For example, one of the widely distributed MPTase domains found in the phage-type polyvalent proteins contains a highly conserved histidine at the end of strand 2 of the conserved core and an aspartate residue three residues downstream from the HEXXH motif, both of which are predicted to project into the active-site pocket (Fig. 3A). In yet another set of polyvalent protein MPTases, the active-site motif is of the form HEXXXH, with four residues instead of the usual three between the conserved histidines and a potential bend in the helix bearing the HEXXXH motif to accommodate the additional residue. A phylogenetic tree of the MPTases of polyvalent proteins suggests that they belong to 12 distinct clades (Fig. 3A; see File S6 at ftp://ftp.ncbi.nih.gov/pub/aravind/polyvalent/polyvalent.html). None of these clades are specifically related to other previously defined clades of zincin-like MPTases, suggesting that they are likely to constitute a distinct group that diversified in the context of polyvalent proteins.

**FIG 4:**
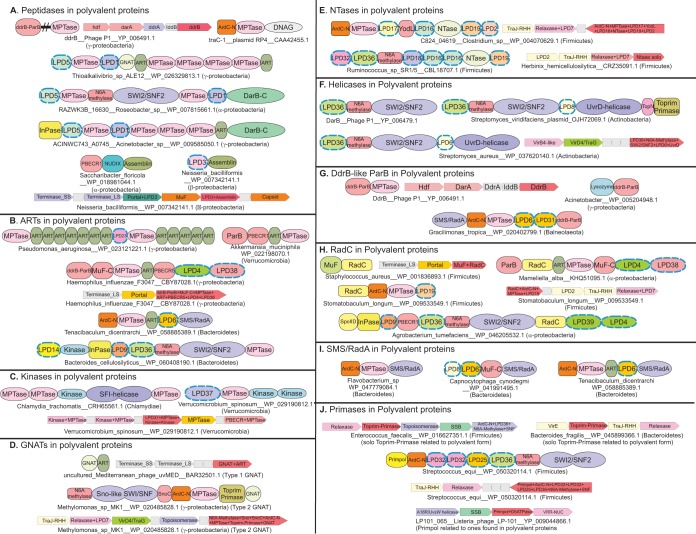
Domain architectures and gene neighborhoods of domains found in polyvalent proteins grouped by the presence of various principal domains or groups of domains, including peptidases (A), ARTs (B), kinases (C), GNATs (D), Pol-β NTases (E), helicases (F), DdrB-like ParB (G), RadC (H), SMS/RadA (I), and Toprim fold and Primpol primases (J). These include only a small sample of the entire diaspora of associations. For the complete set of domain architectures and operons, see ftp://ftp.ncbi.nih.gov/pub/aravind/polyvalent/polyvalent.html. Proteins and gene neighborhoods are shown with their species names and GenBank accession numbers. For gene neighborhoods, the accession number of the gene in dark pink is used. Gene names are shown only for well-studied proteins. Genes in neighborhoods are shown as boxed arrows, with the arrowhead pointing to the 3′ gene. Domains are not drawn to scale.

A clue to the potential function of these MPTases is offered by prior studies on the phage P1 DdrB protein (Fig. 4A) encoded by the *darA* operon. This protein has been shown to be proteolytically processed into a 76-kDa (∼700-aa) polypeptide before being packaged into the phage head (51, 52). On the basis of the C-terminal MPTase domain that we identified in this protein, it is likely that the domain catalyzes the autoproteolysis of the protein before packaging. Although it has not been shown whether the N- or C-terminal fragment is incorporated into the phage head, we predict that it is likely to be the N-terminal fragment with the DdrB-like ParB domain (see below). In contrast to phage P1 DdrB, which is cleaved before encapsidation, the bacteriophage N4 virion RNAP, which also contains an MPTase domain (Fig. 1B), is packaged as an unprocessed protein even though the region encompassing the MPTase domain is required for encapsidation (53, 54). Similarly, the ArdC protein of IncW plasmid pSa and the TraC1 primase of IncP plasmid RP4 (Fig. 4A), both of which contain a C-terminal MPTase, are transferred as full-length polypeptides during the conjugation process (16, 31). These examples suggest that the MPTase domains in polyvalent proteins might also function in processing events other than prepackaging processing. In phage N4 virion RNAP-like polyvalent proteins, the MPTase domain might autoproteolytically process large proteins once they are inside the phage head to enable them to be suitably accommodated and/or readied for release during invasion. Alternatively, as suggested by the plasmid polyvalent proteins, such MPTase domains might also act after being injected into the host cell. In such cases, they could again autoproteolytically release specific domains of the polyvalent protein (e.g., the C-terminal Toprim primase domain seen in TraC1) or convert the inactive polyvalent protein into active products once inside the host cell. The diversity of enzymatic mechanisms of the domains combined into the polyvalent proteins indeed supports this scenario. Finally, it is also possible that some of the MPTase domains act as effectors that proteolytically target host proteins. Both of these functions are compatible with previously reported MPTase domains in conflict systems; they function either as autoproteolytic agents to release other effector domains, as seen in the Photorhabdus virulence cassette systems (20), or themselves act as effectors, as seen in polymorphic toxin systems (10, 46, 47).

The other peptidase superfamilies, i.e., the assemblin-like, DD, and LonC peptidase superfamilies, are rather limited in their distribution. In contrast to the MPTase domains, the assemblin-like peptidase is always found at the C termini of the polyvalent proteins (Fig. 4A). Given the previously described role of the assemblin-like peptidases in the processing of proteins during virion assembly (55, 56), it is likely that they play the same role when found in polyvalent proteins. The DD peptidase is likely to be involved in breaching of the cell wall (see below).

### ARTs.

ARTs use NAD^+^ as a substrate to catalyze the transfer of one or more ADP-ribose moieties to diverse targets, resulting in N-, O-, or S-glycosidic linkages with the 1′' position of ribose (57). The substrate range of ARTs includes a variety of side chains and terminal positions in proteins, nucleic acids, and nucleotides. Previous sequence and structural analyses showed that the primary diversification of ARTs occurred in bacterial and viral biological conflict systems (41). ARTs are by far the most common predicted protein-modifying enzymatic domains found in polyvalent proteins. They are prototyped by the one found in the polyvalent protein from Roseovarius Plymouth podovirus 1 (accession no. CBX87992.1) along with a phage N4-like RNAP module (Fig. 1B). We found that this family of ART domains has an active-site configuration with a histidine (H) in strand 1, a tyrosine (Y) in strand 2, and an aspartate (D) in strand 5. Identification of distinct shared sequence features allowed us to group these with a clade of ART domains, the Tox-ART-HYD2 clade we had previously described in polymorphic toxin systems (10), and a novel class of toxin-antitoxin systems (41, 48). ART domains found in polyvalent proteins are both (i) of phage provenance and (ii) encoded by the conjugation locus of conjugative transposons and plasmids in one or multiple copies per protein in a wide range of architectural contexts (Fig. 4B). The most dramatic instance is a protein from Pseudomonas aeruginosa, a prophage-derived protein with 12 copies of the domain in the same protein (accession no. WP_023121221.1; Fig. 4B).

A model of the action of ARTs in polyvalent proteins is offered by T4 Alt, which is a prototype of a distinct clade of ART domains, the Alt/VIP2 clade that also contains the paralogous ARTs from phage T4, ModA and ModB. These are distinguished from those found in polyvalent proteins by an active site composed of arginine (R), serine (S), and glutamate (E) residues and are grouped in the larger R-S-E-2 clade of ARTs (41); nevertheless, they catalyze similar reactions. Members of this family are present in several T4-like bacteriophages and show conserved genome association with genes for baseplate proteins corresponding to their late translation and packaging into the phage head. While ARTs of this family are not found in genuine polyvalent proteins, they are known to be packaged into the virion, as predicted for the former. Moreover, Alt, unlike its paralogs ModA and ModB, is injected into the host from the phage head along with the viral DNA (21, 22). Thereafter, it ADP-ribosylates host proteins, such as the α subunit of the RNAP, commandeering the host RNAP for viral transcription. The Tox-ART-HYD2 clade, which contains the ART domains from polyvalent proteins, in turn, is related to a wide range of protein-modifying ARTs, such as poly(ADP-ribose) polymerases, Gig2, and cholix/diphtheria toxins (21, 41). Hence, we postulate that those in polyvalent proteins are likely to modify host proteins to favor the establishment and replication of invasive DNA elements. The fusion of the Tox-ART-HYD2-like ART to the N4-like RNAPs in some caudoviruses (Fig. 1B) suggests that it might function similarly to T4 Alt by modifying host proteins in conjunction with the initiation of viral transcription.

### Protein kinases.

Polyvalent proteins feature a distinctive monophyletic group of kinase domains of the serine/threonine/tyrosine kinase superfamily. The sequence conservation pattern and the corresponding predicted structural elements indicate that these domains are united by the unusual loss of the entire C-terminal subdomain typical of protein kinases (58) while leaving the conserved active-site residues involved in ATP binding and phosphotransfer intact (59) (see File S2 at ftp://ftp.ncbi.nih.gov/pub/aravind/polyvalent/polyvalent.html). They also contain unique distinguishing features such as a glutamate N terminal to strand 2 associated with the ATP-binding site, a conserved glutamine in the last strand of the N-terminal α+β subdomain, and an insert between the conserved histidine and aspartate residues involved in phosphotransfer (see File S2 at the URL mentioned above). This type of kinase domain is present primarily in polyvalent proteins of (pro)phages of the Verrucomicrobia-Chlamydia and Bacteroidetes lineages of bacteria. The latter additionally contain stand-alone versions of this distinct kinase domain. In Verrucomicrobium spinosum, the kinase-containing polyvalent proteins are clustered in two loci. In the vicinity of the gene encoding the large polyvalent protein, which is likely to be the primary locus, there are several smaller open reading frames coding for other domains typical of polyvalent proteins (Fig. 4C). These are reminiscent of the cassettes seen in polymorphic toxin systems, where the cassettes are recombined into the main locus to generate new variations (10), raising the possibility of similar recombinational variability in this system (Fig. 4C). A precedent for the action of these enzymes is offered by the phage T7 protein kinase, which phosphorylates multiple host proteins, including several translation factors (60). The kinase domains from polyvalent proteins, while greatly structurally modified, are closer to protein kinases in the conserved core they retain. Hence, it is quite possible that, like the T7 kinase, they phosphorylate specific proteins to alter their function as part of the biological conflicts in which they are deployed (60).

### GNATs.

GNATs transfer acetyl/acyl moieties from acetyl/acyl coenzyme A to NH_2_ groups in a diverse array of substrates, from small molecules to polymers, such as proteins, DNA, and carbohydrates (61, 62). Two distinct families of GNATs are detected in polyvalent proteins, and all of them are found in distinct genomic and phyletic contexts. One of them has so far been seen only in gammaproteobacterial (pro)phages and in an uncultured Mediterranean phage (accession no. BAR32501.1) (Fig. 4D). Solo members of this family of GNATs are also seen in Firmicutes and Actinobacteria. In polyvalent proteins, this GNAT domain typically has the above-described ART domain as a neighbor in the same polypeptide. The second family, which is found in Firmicutes, Actinobacteria, and Proteobacteria, is encoded by mobile conjugating transposons or plasmids utilizing a T4SS-like delivery system. Solo versions of these are also found in certain mycobacteriophages along with other late structural genes (e.g., gp113 from Mycobacterium phage Alice), suggesting deployment in the capsid like the polyvalent proteins. Both of these families, though not sister clades, belong to the larger clade of RimI-like GNATs (prototyped by the eponymous ribosomal protein acetyltransferase [61, 62]), suggesting that they are likely to modify protein substrates. The presence of solo versions of both families suggests that the two were probably independently acquired by polyvalent proteins of phage origin.

### Pol-β superfamily NTases.

These NTase domains are found primarily in polyvalent proteins from firmicute mobile elements. They are usually fused to an ArdC-N domain (see below) and found in the context of genes coding for T4SS-like DNA transfer systems of conjugative transposons or plasmids (Fig. 4E). In addition to polyvalent proteins, these NTase domains are also found as solo proteins in related firmicute mobile elements. Structure prediction based on sequence conservation revealed that this NTase domain is a minimal version of the Pol-β fold consisting of four strands and three helices (Fig. 3B) (63). They are most closely related to the NTox45 toxin domain described earlier in polymorphic toxin systems (10). Further, they are also more distantly related to the minimal NTase domains found in type II TA systems, where they appear to be the antitoxin linked to a HEPN domain RNase toxin (6, 64). The relationship to the predicted antitoxin NTases of the type II TA systems suggests that they might act similarly by transferring a nucleoside monophosphate to a protein substrate (6, 64).

### Catalytic domains in polyvalent proteins predicted to operate on nucleic acids.

Given the effectiveness of damage to genomic DNA and various RNAs, particularly those associated with translation, in crippling a biological system, enzymes targeting nucleic acids are the mainstay in biological conflicts on both the side of the host and that of invasive elements (9, 10, 41). The resulting arms race has selected for a wide arsenal of enzymes, both those that damage nucleic acids and those that protect against or help specifically target such damage across diverse conflict and counterconflict systems (9, 10, 65). A second set of enzymatic domains that operates on nucleic acids directly facilitate the replication or transcription of the invasive genome. We were able to identify several catalytic domains predicted to operate on DNA or RNA in polyvalent proteins, suggesting that such interactions are an important aspect of biological conflicts occurring in the early phase of invasion by phages and mobile elements.

### The DNA methylase-helicase dyad.

The third most prevalent domain in polyvalent proteins is an MTase domain that is nearly always fused at its C terminus to a superfamily II (SF2) helicase and at its N terminus to a previously uncharacterized domain, LPD36, found only in polyvalent proteins. The archetypal member is the DarB protein from phage P1/P7, which is involved in antirestriction (32) (Fig. 4F). Through sequence and structural analyses of the MTase domains in polyvalent proteins, we found that they are mostly monophyletic and belong to the EcoKI/TaqI-like family of DNA adenine MTases (66, 67). They share with other members of this family a conserved helix N terminal to the core MTase domain that further is capped by an N-terminal TP motif that interacts with an asparagine in the active site occurring as part of an NPP(Y/F) motif. This observation suggests that they are likely to be adenine N6-MTases (gamma class MTases) (66, 67) (see File S2 at ftp://ftp.ncbi.nih.gov/pub/aravind/polyvalent/polyvalent.html). We found that the N-terminal LPD36 domain is likely to adopt a fold with four α helices and is distinguished by a characteristic GXGU motif (where U is G, A, or S) between the second and third conserved helices. Although profile searches did not retrieve any relationship to known domains, its position is reminiscent of the α-helical HdsM-N domain that is found N terminal to the EcoKI-like methylase found in type I restriction enzymes. This observation, together with the above-noted motif, which is suggestive of nucleotide binding, suggests that the LPD36 domain might be involved in base recognition and flipping, as seen in equivalent N-terminal domains of other adenine MTases (66, 68).

The SF2 helicase-like module associated with the C terminus of the adenine MTase belongs to a vast group of enzymes performing ATP-dependent helicase or translocation activity on various nucleic acid polymers. Analysis of the sequence conservation patterns revealed that there are two major families of SF2 helicase-like domains in polyvalent proteins, both of which are specifically related to the SWI2/SNF2 ATPase clade. Like the classical SWI2/SNF2 ATPases, these families from polyvalent proteins also possess a multihelical insert after strand 4 (the strand following the Walker B motif) of the first of the two nucleoside triphosphatase (NTPase) domains typical of helicase-like modules (69). They also share a multihelical insert after strand 1 of the second NTPase unit. In a few instances, polyvalent proteins have a Strawberry notch-like SWI2/SNF2 ATPase (69) distinguished by its unique C-terminal winged helix-turn-helix domain (e.g., Methylomonas SnoC-wHTH; accession no. WP_020485828.1; Fig. 4D). Even outside polyvalent proteins, these SWI2/SNF2 ATPases have been found widely coupled to adenine MTases, in the same polypeptide or via a conserved gene neighborhood, in phages, plasmids, and conjugative transposons (9). Similar coupling of helicase-like modules and MTases is also found in various R-M systems, including classical type I and III R-M systems (9, 66, 70). More broadly, other families of prokaryotic SWI2/SNF2 ATPases, including RapA/HepA, which is involved in release of the RNAP holoenzyme from the posttranscription/posttermination complex (71, 72), and the SWIM domain-associated SWI2/SNF2 ATPases (SsoRad54) (73), are also widely present in mobile systems in phages (70, 74). This suggests their monophyly and explosive diversification in the context of these conflict systems, followed by transfer to eukaryotes on multiple independent occasions (75).

The archetypal member of this group, DarB, is a structural component of the phage P1 virion head (Fig. 4F) (51) and has been shown to protect the phage DNA in *cis* against attack by EcoK1-like type I restriction enzymes (32). The strong association of SWI2/SNF2 ATPases with a particular type of adenine methylase suggests a unique biochemical partnership of these domains in the context of DNA transfer. On the basis of these observations, it appears that the injected DNA is modified by the DarB-like methylase but strictly requires the ATPase activity of the SWI2/SNF2 proteins. Studies on the ATPase activities of SWI2/SNF2 proteins suggest that they translocate along the dsDNA minor groove in an ATPase-dependent manner without separating the duplex DNA (73, 76–78). Its combination with the methylase suggests a mechanism similar to that of type III restriction enzymes, where a distinct SF2 helicase fused to a restriction enzyme (Res subunit) combines with a methylase (Mod subunit) and translocates along the DNA until it collides with a second such pair, at which point the DNA is methylated or restricted, depending on the prior methylation state of the DNA (79). A similar mechanism can be conceived for the methylase-helicase fusion of the polyvalent proteins, which might bind the linear DNA of the element injected into the recipient cells and methylate it to discriminate invasive DNA from host DNA or protect the former against restriction. In a few actinomycetes, prototyped by the gigantic protein OJH72069.1 (12,242 aa) encoded by a Streptomyces viridifaciens plasmid, a UvrD-like superfamily I helicase (80) cooccurs with the above SWI2/SNF2 helicases (Fig. 4F). These UvrD helicases are closely related to their cellular counterparts, and the polyvalent proteins in which they are present appear in contexts very similar to those of conjugative transfer systems. The cellular versions of these helicases are components of the nucleotide excision repair machinery and act on ssDNA (80). On the basis of this precedent, we suggest that in the invasive elements, they play a role similar to the above-described SWI2/SNF2 helicases but instead act on the ssDNA transferred by the conjugative plasmid/element.

### Novel ParB-like DNases.

**FIG 5:**
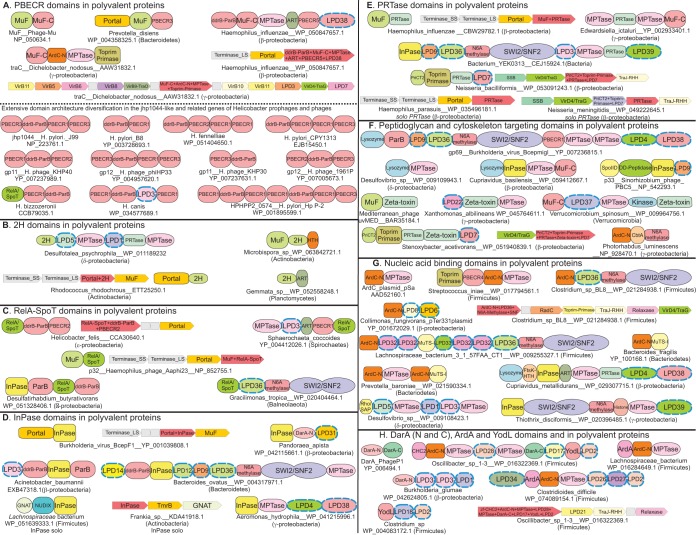
Domain architectures and gene neighborhoods of domains found in polyvalent proteins grouped by the presence of various principal domains or groups of domains, including PBECR (A); 2H (B); RelA/SpoT (C); inorganic pyrophosphatases (D); phosphoribosyltransferases (E); peptidoglycan and cytoskeleton-targeting domains (F); nucleic acid binding domains (G); and DarA, ArdA, and YodL domains (H). Domain and gene neighborhood designations are as in Fig. 4.

We recovered a distinct family of domains belonging to the ParB/sulfiredoxin (ParB/Srx) superfamily in polyvalent proteins, which are prototyped by the version found in the phage P1 DdrB protein (hence, we term them DdrB-like ParB domains). DdrB-like ParB domains have an α+β fold with four strands and two conserved helices in common with other members of the ParB/Srx superfamily (81). They also contain a highly conserved arginine residue lodged in the active-site pocket typical of this superfamily, which is the site of both metal-dependent DNase activity and ATP-binding/hydrolysis (81). Additionally, they display lineage-specific residues, including an aspartate/asparagine at the end of strand 2 (Fig. 3C). Further, they contain a unique helix between the terminal helix and strand which bears a conserved tyrosine that might be important for activity. Barring a few exceptions, DdrB-like ParB domains, either in solo form or in polyvalent proteins, are found in phages or prophages, where they are often in the context of late genes typically next to virion structural proteins (Fig. 4G and B and 5A). Studies on phage P1 showed that a processed form of DdrB (probably by autopeptidase activity of its C-terminal MPTase domain) is incorporated into the phage head (51, 52). In polyvalent proteins from prophages of Acinetobacter (e.g., accession no. WP_005204948) the DdrB-like ParB domain is fused to a lysozyme domain in the context of late phage genes, supporting the idea that it is injected along with breaching of the host peptidoglycan by the latter domain. In the exceptional instance of Gracilimonas (accession no. WP_020402799), a DdrB-like ParB domain is associated with an ArdC-N domain that is normally associated with T4SS-like DNA transfer systems (see below). However, the genomic contexts do not reveal any further connections to mobile elements or phages, suggesting that it might have been acquired by the host as defense against invading elements (see below).

Recent studies on ParB domains in several distinct contexts have shown them to be DNases and/or nucleotidases. For example, in the type IV GmrSD-like restriction system, the ParB-like domain (GmrS) is fused to an HNH domain and is likely to function as a UTPase and a nonspecific nuclease (82, 83). In Bacillus cereus Bce_0392 (ParB-methylase), the ParB domain, which is fused to an adenine methylase, was shown to possess nonspecific nicking endonuclease activity (84). In the Osa protein, the ParB-like domain was shown to possess ATPase and DNase activities (81). Similar nuclease/NTPase activities have been reported for ParB domains in sulfiredoxin and other ParB domains (81, 85, 86). One aspect that is common across these studies is that the nuclease activity is largely sequence nonspecific; further, it might be coupled to an intrinsic NTPase activity that might regulate the nuclease activity. In cellular contexts, ParB proteins function in close conjunction with the ATPase ParA in the context of chromosome partitioning (87). However, such an association with ParA-like proteins is absent in the case of polyvalent proteins, suggesting that the ParB domains in polyvalent proteins are likely to be NTP-regulated DNases with roles unrelated to chromosome partitioning. It is possible that their nicking/DNase activity assists the early phase of DNA replication of the invasive element. Alternatively, they might degrade host DNA, facilitating replication arrest of the host, as has been reported in phage infections (88). In a similar vein, these domains could also function like the Osa protein by degrading rival or superinfecting invasive elements (81). While ParB-like DNases have been recorded along with other nuclease domains in multiple prokaryotic nucleic acid-targeting conflict systems (e.g., polymorphic toxins [10], restriction systems [83], Dnd systems involving phosphorothioate modification of the DNA backbone [89], and plasmid sexual conflict systems [81]), they are not displaced or accompanied by other DNase domains in polyvalent proteins. Hence, it is more likely that specific sensing/degradation of one or more NTPs by the DdrB-like ParB domains is a key function required in the early infection strategies of the phages that display them.

### The RadC domain.

The RadC domain is a widely distributed prokaryotic clade of JAB domains and is observed in polyvalent proteins from mobile DNA elements and prophages of diverse prokaryotes (Fig. 4H). Whereas the currently biochemically characterized versions of the JAB domain are C-terminal peptidases of ubiquitin and related proteins, Escherichia coli RadC has been implicated in DNA repair (90), with some dispute (91). Further, we have shown that the RadC family of JAB domains, in contrast to the classic JAB domains, are unlikely to contain the groove that accommodates the ubiquitin tail (46). On the basis of contextual information such as fusions to HhH, ArdC-N, and DinG/RAD3-like superfamily II helicases, which display a positional equivalence to other nucleases, we hypothesized that the domain might be a nuclease or nucleic acid-processing domain (46). Moreover, the JAB domain displays a structural fold that is otherwise seen primarily in enzymes operating on nucleic acids or free nucleotides (i.e., the deaminase fold). This suggests that the RadC domain from polyvalent proteins is probably involved in the processing of nucleic acids or nucleotide substrates.

### The SMS/RadA domain.

The SMS/RadA domain, found in polyvalent proteins of Bacteroidetes species, belongs to the RecA-like superfamily of P-loop NTPases, within which they are sister lineages of the KaiC family of proteins (92). Domain architectures suggest that they are seen mainly in polyvalent proteins of conjugative elements or plasmids (Fig. 4I). SMS/RadA proteins are implicated in the resolution of recombinational intermediates such as Holliday junctions (93, 94). In light of these observations, in the context of polyvalent proteins, they might be involved in the initial manipulation of the incoming DNA, such as during circularization or integration into the host genome.

### Primases and RNAPs.

Two unrelated primase domains, namely, the Toprim-type (e.g., DnaG primases) and the archaeoeukaryotic primase fold Primpol (often with their associated domains C2HCZn cluster, PriCT2, and PriCT1), and the coliphage N4-like virion RNAP modules are widely found across polyvalent proteins (Fig. 1B, 4F and J, and 5A, E, F, and G). Our analysis showed that several profiles labeled as generic Toprim domains in the Pfam database (Toprim_2, Toprim_3, and Toprim_4) are actually versions of the catalytically active Toprim domain of DnaG-like primases from mobile elements. These Toprim domains are unified by a synapomorphic DaN– motif (where “a” is aromatic and “–” is an acidic residue) in the helix before strand 4 (see File S2 at ftp://ftp.ncbi.nih.gov/pub/aravind/polyvalent/polyvalent.html) (95). The Toprim primases are always encoded by mobile elements with T4SS-like transfer systems and never by phages. The Toprim primase modules from polyvalent proteins are specifically related to those found as stand-alone versions in other conjugative elements (Fig. 4J). Further, phylogenetic analyses suggest that primase domains in polyvalent proteins are most closely related to the solo version of the domain in the same phylogenetic group. Those found in Firmicutes are closely related to solo Firmicutes versions, and those from Proteobacteria, correspondingly, are closely related to proteobacterial solo versions. The Primpol domains (96) in polyvalent proteins are likewise found only in conjugative elements (Tn*1549*-like) and are limited to only the Firmicutes and Fusobacteria (Fig. 1C and 4J). These are specifically related to Primpols found in bacteriophages infecting Firmicutes (e.g., Listeria phage LP-101, accession no. AHL18844.1), suggesting a phage source for this domain in the conjugative elements. The above patterns suggest that the primase domains have been independently incorporated into polyvalent proteins on multiple occasions. This provided us with evidence of a strong selective pressure that channels the emergence of these multidomain polyvalent proteins across diverse elements.

In contrast, the N4-like virion RNAPs are found only in N4-like phages or prophages in stereotypic gene neighborhoods with late genes coding for virion components (35) (Fig. 1D). The RNAP module is almost always fused to an MPTase domain and shows some diversity in its domain architectures, with associations with ART, lysozyme, and PBECR domains (Fig. 1B). Examples of both the primases and the N4 virion RNAP modules, respectively, from phages and conjugating elements have been shown to be transferred along with DNA (16, 37, 96, 97). This corresponds to their early role in the life cycle of these elements: the primases prime the replication of the transferred ssDNA in conjugative elements, whereas the virion RNAPs allow the transcription of early phage genes immediately upon invasion.

### PBECR: a novel predicted RNase domain in polyvalent proteins.

The BECR fold of metal-independent endoRNases are commonly observed and extensively diversified effectors across diverse prokaryotic conflict systems such as polymorphic toxin systems, plasmid-encoded bacteriocins, and TA systems (10). Although BECR fold proteins are often extensively elaborated with inserts or show structural modifications, they share a conserved core of an N-terminal helix followed by four or five strands (10, 58). The active-site residues often show much variation, but a commonly observed configuration includes a conserved alcoholic residue (S/T) in strand 4 and histidine in the N-terminal helix (10) (Fig. 3D). We detected a novel clade of the BECR clade present in up to 26% of the polyvalent proteins. These can be further divided into six distinct families of which five share an active-site configuration of a histidine in the N-terminal helix and a C-terminal threonine/serine, as seen in BECR endoRNases like colicin D and those found in diverse polymorphic toxins (10, 58). The sixth contains an arginine instead of a histidine at the same position (Fig. 3D). In addition to their presence in polyvalent proteins, solo versions of these BECR domains are also found in phages; we accordingly designate this clade the phage-BECR (PBECR) domains. The simplest architectures involve a fusion of the PBECR to MuF and portal domains (Fig. 5A), while larger polyvalent proteins with this domain are encoded in the context of virion components, suggesting that they are likely packaged into the virion and injected by the phage into the host (26). The classical bacteriophage Mu protein MuF (gp30; NP_050634.1), a major component of the phage head contains a C-terminal PBECR domain (MuF-C domain, Fig. 3D and 5A) (98). In addition to (pro)phages PBECR domains are also encoded by certain mobile elements with T4SS-like DNA delivery apparatus in both solo and polyvalent proteins (Fig. 5A). PBECR domains show a great diversity of domain architectural contexts in polyvalent proteins. This is exemplified by a remarkable diversification of the domain architectures of orthologous polyvalent proteins (jhp1044 of Helicobacter pylori) across a group of Helicobacter (pro)phages. The basic collection of domains in this polyvalent protein includes four distinct PBECR domains and DdrB-like ParB (Fig. 5A). In non-H. pylori species, we additionally detected RelA-SpoT and LPD3 domains in the same context. However, these individual domains undergo extensive duplications and rearrangements to make up to 50 distinct domain combinations. This extensive mosaicism (99, 100) suggests that these proteins are rapidly evolving because of a dynamic conflict either with the Helicobacter host or with other competing parasitic elements.

Previously characterized BECR domains from conflict systems are often involved in the cleavage of conserved RNA molecules of the host such as tRNA or rRNA (101–103) or, in some instances, ribosome-associated mRNAs directly during the process of translation (104). The well-studied phage BECR fold protein RegB facilitates the phage transcriptional cascade by cleaving early phage mRNAs (104). On the basis of this precedence, one possibility is that the PBECR domains do not cleave host tRNA and rRNA molecules, being dependent on them for translation; instead, they might target host and/or phage mRNA molecules. However, their diversity in polyvalent proteins is suggestive of a direct coevolutionary response to a host RNA-based restriction mechanism. An interesting alternative is that some of them target the CRISPR RNAs of the CRISPR/Cas systems that are deployed against incoming parasitic elements (105). The latter possibility might explain the extensive mosaicism of H. pylori jhp1044, where the PBECR domains in these proteins are possibly in an arms race with the adaptive CRISPR system, leading to the observed diversity of domain architectures and sequences. This presence of an inactive PBECR domain in the poorly characterized phage Mu MuF protein (gp30) (Fig. 3D and 5A) also predicts a previously unknown role for this domain, probably via RNA binding, whereas the catalytically active PBECR domains of orthologs of phage Mu MuF are likely to function as RNases.

### The 2H phosphoesterase domain.

The 2H phosphoesterase domains catalyze a related set of reactions, namely, as processing enzymes of ends of RNAs with 2′-3′ cyclic phosphate linkages produced by metal-independent RNase attacks, cyclic nucleotides with 2′-3′ cyclic linkages, and polynucleotides with 2′-5′ linkages, and as RNases which generate ends with cyclic 2′-3′ phosphates (65, 106). When fused to or associated with ATP-grasp RNA ligases, they process 2′-3′ cyclic phosphate ends to allow ligation of RNAs damaged by metal-independent nuclease attacks. 2H domains are present both in polyvalent proteins and as standalone domains in several phages. However, despite the widespread presence of ATP-grasp RNA ligases in phages (e.g., phage T4 RNA repair system) (65, 107, 108), we never observed the specific versions of 2H domains found in polyvalent proteins or their solo counterparts associating with RNA ligases. On the basis of this observation, we propose that these 2H domains are more likely to function either as RNases comparable to the eukaryotic 2H protein Usb1/Mpn1 (106) or like the versions found in eukaryotic RNA viruses that degrade 2′-5′ oligoadenylate (109). The former suggestion is consistent with the fusion of 2H domains to MuF or portal domains in architectures comparable to the fusions of the PBECR domains to MuF or portal domains (Fig. 5B). We have recently reported that prokaryotic counterinvader systems, including several of the CRISPR/Cas systems, are activated by cyclic nucleotides, 2′-5′ oligoadenylate, or related nucleotides (65). Hence, consistent with the latter study, these 2H domains could also help degrade nucleotides generated by the nucleotide-activated counterinvader systems.

### Domains involved in nucleotide metabolism.

Recent studies have shown that nucleotides and other small molecules derived from them are signaling agents at the center of several intergenomic conflicts in both prokaryotes and eukaryotes (3, 4, 110). For example, the Ter system is predicted to synthesize and/or sense a nucleoside-derived compound with a potential role in antibacteriophage resistance (3). CRISPR/Cas systems possess both a nucleotide-generating enzyme, the CRISPR polymerase (Cpol), and nucleotide recognition domains such as CARF and WYL, implying that nucleotides are intimately involved in the regulation of the CRISPR/Cas defense response (4, 110). Similarly, several nucleotide-centric systems related to the animal 2′-5′ oligoadenylate and cyclic dinucleotide-activated responses have been uncovered as regulators of counterinvasive element defenses in prokaryotes (110). In this regard, the above prediction of the 2H phosphoesterase domains in polyvalent proteins as potential counters for host conflict-related nucleotide signaling systems is of note. Additionally, we found four distinct enzymatic domains in polyvalent proteins that might play further roles in nucleotide metabolism.

### RelA-SpoT and GGDEF NTase domains.

While not very widespread, the presence of RelA/SpoT (Fig. 5C) and GGDEF (Fig. 1C) domains in polyvalent proteins suggests that the invasive elements might also use (cyclic) nucleotide signals. The GGDEF domains are known to synthesize cyclic nucleotides such as cyclic diguanylate and diadenylate (111, 112). The deployment of such a cyclic nucleotide-generating enzyme might help in either interference with host response utilizing similar nucleotides or in the activation of a response against rival elements while integrated/residing in the host genome. The RelA/SpoT domain, which is more widely distributed than the former in polyvalent proteins (Fig. 5C), is an NTase domain of the Pol-β superfamily that synthesizes the alarmone(p)ppGpp or related nucleotides (63, 113). Phylogenetic analysis suggests that the polyvalent proteins have independently acquired the RelA/SpoT domain in (pro)phages of Spirochaetes and epsilon Proteobacteria. RelA/SpoT domains are also fused to the MuF domain in phage/prophages and in MuF-based toxin delivery systems (10) (Fig. 5C). Interestingly, RelA/SpoT domains are also deployed as effectors in other toxin systems such as the polymorphic toxin systems and type II TA systems, suggesting that alarmone synthesis is a commonly used weapon in biological conflicts (10). The alarmone has pleiotropic effects on the cell, such as alteration of the transcriptional activity of RNAP and reduction of DNA replication and protein synthesis (114). The presence of this domain in polyvalent proteins suggests that phages deploying this domain might trigger an alarmone-like response to potentially shut down or alter the host transcriptional and translational profile.

### The InPase domain.

“Inorganic pyrophosphatase” (InPase) domains belonging to the so-called type I InPase family (115) and, containing an OB fold catalytic domain (116), are incorporated in diverse domain architectural contexts in polyvalent proteins (Fig. 4 and 5D). Their active site is composed of four conserved acidic residues that coordinate two metal ions that activate a water molecule, followed by proton abstraction by one of the conserved acidic residues (117). All previously structurally characterized members in the PDB database contain an additional β hairpin between the first and the second strands of the core fold. However, InPase domains from polyvalent proteins are distinct in lacking this β hairpin insert, suggesting a monophyletic origin for this group of InPase domains (see File S2 at ftp://ftp.ncbi.nih.gov/pub/aravind/polyvalent/polyvalent.html). Analysis of the genome contexts of these proteins suggests that they are present mainly in (pro)phage polyvalent proteins and are likely to be packaged into the virion (Fig. 5D, F, and G). Consistent with this, versions of the domain are also fused to the portal domain (e.g., YP_001039808.1/BcepF1.124 from Burkholderia virus BcepF1), suggesting that they might be injected into the host during infection. Related InPase domains are encoded in the neighborhood of or are fused to the NUDIX and GNAT domains (e.g., Lachnospiraceae WP_051639333.1), while others from actinobacteria (e.g., Frankia, KDA41918.1) are linked in neighborhoods with the tunicamycin (a nucleotide derivative [118]) resistance protein TmrB (a member of the P-loop NTPase superfamily). This suggests a possible role for these InPase domains in the processing of phosphoester linkages in a nucleotide-like substrate (119). Thus, the domain might specifically target nucleotides that are made in response to infection by parasitic elements. Alternatively, the InPase domain could directly target PP_i_ released from the nucleotide-processing reactions. Several reactions, such as those catalyzed by NTases and polymerases, are inhibited by PP_i_, a product of their catalysis. Eukaryotic type I InPases are components of the NurF chromatin complex, wherein they are proposed to clear PP_i_ to assist replication or transcription (120). This is similar to the proposal that the phosphoesterase domains found in polymerases and NTases clear the inhibitory PP_i_ (121). Thus, the InPase associated with polyvalent proteins could also clear PP_i_ to improve the efficiency of early events such as replication or transcription of invasive elements.

### Phosphoribosyltransferase (PRTase) domain.

Classic members of the PRTase superfamily catalyze the replacement of the diphosphate in 5-phospho-α-d-ribose 1-diphosphate (PRPP) with a purine or pyrimidine base or an NH_2_ group along with anomeric inversion of the ribose ring (122). The PRTase domain is found primarily in polyvalent proteins of elements from Proteobacteria and Verrucomicrobiae. It is predominantly found in (pro)phage polyvalent proteins, with the simplest multidomain architectures showing fusions to the MuF domain (Fig. 5E). Less frequently, they are seen in polyvalent proteins of conjugative elements. Related solo PRTase domains are also widely found in both contexts (Fig. 5E). Analysis of the sequence conservation patterns and domain architectures reveals that the PRTases were independently incorporated into phage and plasmid polyvalent proteins. Nevertheless, all PRTases from invasive elements form a distinct group within the PRTase superfamily; besides the characteristic catalytic DD motif, they share several other unique sequence features (see File S2 at ftp://ftp.ncbi.nih.gov/pub/aravind/polyvalent/polyvalent.html). Moreover, they share a highly conserved arginine in the loop after the second strand with the ComFC-like PRTases, which have been previously implicated in competence-related DNA uptake (123, 124).

The role of the PRTase domain in these selfish elements is somewhat enigmatic. Several parallels are suggested by other PRTase domains; like the classic PRTase domains, they could be involved in the synthesis of a nucleotide from a free base and PRPP. Indeed, the production of such a nucleotide has been proposed for the two PRTases found in the Ter system, which plays a role in immunity against bacteriophage and plasmid invasion (3). Besides nucleotide metabolism, versions of the PRTase domain are found in the competence system (e.g., ComFC), where, along with a helicase (ComFA), they play a role in DNA uptake (3, 123, 124). In a related system, the PRTase domain is fused to a distinct SFII helicase (125, 126) that appears to be functionally coupled with another protein implicated in DNA uptake, DprA. This system has been implicated in a DNA repair or an SOS-like response (125, 126), which could be triggered by invasive DNA. Further, the classic PRTases are also known to bind single-stranded RNA (127), raising the possibility of a similar direct interaction with ssDNA. Taking these findings together, we propose that the PRTase domain found in these selfish elements might have a role in the production or sensing of a nucleotide in the context of DNA entry. One possibility is that they help invasive elements and thereby target rival elements that might access the same cell.

### Enzymes targeting peptidoglycan and cytoskeletal proteins.

The peptidoglycan polymer with a polysaccharide backbone and oligopeptide “cross-links,” which constitutes the bacterial cell wall, poses a significant barrier for invasive elements trying to access the host cell. Polyvalent proteins frequently possess lysozyme domains, which cleave the polysaccharide linkages in peptidoglycan (Fig. 1C, 4G, and 5F). In the case of phages, lysozyme domains have been observed as domains in tail, portal, sheath, and capsid proteins (128, 129). Hence, we postulate that, as in these cases, the versions found in polyvalent proteins are likely to help breach the peptidoglycan barrier at the time of invasion. In a small number of instances, polyvalent proteins also possess the SpoIID-like transglycosylase domain (Pfam code PF07486), which also cleaves peptidoglycan but belongs to a fold distinct from the lysozyme domain (130) (Fig. 5F). We also found a relatively limited subset of polyvalent proteins with DD-peptidase domains that are known to hydrolyze the linkages between d-amino acids unique to the oligopeptides in peptidoglycan (Fig. 5F) (131). These too, on being incorporated into the virion, are likely to assist in injection of the DNA across the peptidoglycan barrier by targeting its peptide linkages. Another domain acting on the peptidoglycan polymer found in polyvalent proteins of conjugative elements from Verrucomicrobium and Proteobacteria and fused to the MuF domains in phages is the zeta-toxin-like kinase that inhibits cell wall synthesis by phosphorylating precursor sugars, resulting in cell lysis (132) (Fig. 5F). In these elements, they might weaken the cell wall and facilitate the assembly of the conjugative apparatus or injection of phage DNA. The CbtA/YeeV toxin is found in TA systems (YeeU-YeeV) and inhibits the nucleotide-dependent polymerization of the cytoskeletal proteins MreB and FtsZ (133). In polyvalent proteins, these are present in conjugating elements only. It is possible that they function by inhibiting or delaying cell division, allowing the element to complete its replication.

### Noncatalytic and miscellaneous uncharacterized domains found in polyvalent proteins.

The above-described domains are the primary catalytic domains for which different levels of biochemical function prediction could be made from a total of about 131 domains found in polyvalent proteins. Below we detail the remaining domains that nucleate around the above domains, which can be generally grouped into three types, (i) various noncatalytic domains with predictable function, (ii) previously defined domains with poorly characterized function, and (iii) newly defined large polyvalent-protein-associated domains (LPDs).

### Nucleic acid binding domains ArdC-N, MutS-I, FtsK, and Rho-N.

Nucleic acid-binding domains constitute the largest group of noncatalytic domains in polyvalent proteins for which clear functional predictions could be adduced. The most prevalent of these (∼26% of the polyvalent proteins have a copy) is one prototyped by the N terminus of the plasmid pSa antirestriction protein ArdC (here ArdC-N; accession no. AAD52160.1). It is overall the second most common domain in polyvalent proteins. Previous studies have suggested that the ArdC-N domain binds ssDNA (16, 31, 37). Using sensitive sequence analysis methods, we have now unified it to the DNA-binding BHD_1, BHD_2, and BHD_3 domains of XPC/Rad4 (134) and the Trypanosoma Tc38 family proteins, which are DNA-binding proteins associating with the unique mitochondrial DNA circles of these organisms (135; A. M. Burroughs, L. M. Iyer, S. Anand, and L. Aravind, unpublished data). The core of this domain comprises one or two N-terminal α helices followed by four β strands, arranged as a nested-hairpin structure. Examination of the XPC/Rad4 structure (PDB code 2QSH) indicates that the nested hairpin structure is critical for binding of ssDNA, suggesting that this might be a conserved feature of ArdC-N domains (134, 136–138). In polyvalent proteins, the ArdC-N domain is often at the N terminus and is most frequently associated with conjugative elements. One of the most common domains cooccurring with ArdC-N in the same polypeptide is the MPTase domain (∼70% of cases), suggesting that the former might be released by the autoproteolytic action of the MPTase domain. The MutS-I DNA-binding domain is found in polyvalent proteins from the Bacteroidetes and Firmicutes lineages. The archetypal version of this superfamily of domains is the N-terminal domain of the mismatch repair ATPase protein MutS. In MutS, this is known to specifically bind mismatched single-stranded regions of DNA much like the Rad4 BHD domains (136, 139, 140), suggesting that, in polyvalent proteins, they might function comparably to the ArdC-N domain. However, we observed that in ∼80% of the cases, the MutS-I domain in polyvalent proteins cooccurs with an ArdC-N domain, suggesting that they are not mutually exclusive and might recognize distinct features of ssDNA. The presence of the MutS-I domains in polyvalent proteins is the only instance to date where the MutS-I domain occurs outside the MutS protein, which is found across most major bacterial lineages. This suggests that the MutS-I domain in polyvalent proteins was likely derived from the classical MutS protein.

The precedence of ArdC and TraC1 (31), which are transferred along with DNA, suggests that, in most instances where these ssDNA-binding domains are present, the polyvalent proteins are transferred with DNA into the recipient cell bound to the genomic substrate. Given the previously noted role of ArdC (31), we propose that ArdC-N′s wide distribution in conjugative elements possibly results from it protection of the single-stranded genome of the invasive element from type II restriction enzymes during DNA entry. Additionally, the specialized N-terminal location of the ArdC-N domain suggests that it might function as a “header” domain that couples the rest of the polyvalent protein as it is being delivered via the conjugation apparatus. The less frequent occurrence of MutS-I domains suggests that it might potentially recognize shorter ssDNA stretches associated with the replicating element shortly after entry into the host cell.

In addition to the above, we also recovered multiple infrequently occurring DNA-binding domains such as the FtsK wHTH domain (e.g., Cupriavidus WP_029307715), the Rho-N/SAP HEH fold domain (e.g., Desulfovibrio WP_009108423), other HTH domains, and a histone fold (e.g., Thiothrix WP_020396485) domains (Fig. 5G). While rare, all of these domains are predicted to bind dsDNA, suggesting that they have a role in phages with dsDNA or postreplication regulatory functions in conjugative elements.

### The DarA, ArdA, and YodL domains.

While the DarA, ArdA, and YodL domains occur in previously studied proteins, their precise biochemical activities remain obscure. The first of these is defined by the N-terminal domain of the DarA protein and the sole domain in the hdf protein, both from phage P1 (Fig. 4G and 5H). Structure prediction revealed that DarA-N has an α+β fold with a conserved aspartate and an asparagine residue followed by a basic residue (NX+ motif, see File S2 at ftp://ftp.ncbi.nih.gov/pub/aravind/polyvalent/polyvalent.html). In phage P1, DarA is known to be proteolytically cleaved and incorporated into the phage head (51, 52). Its homolog, the hdf protein, has also been suspected to be incorporated into the phage virion (51, 52). DarA has been implicated in the countering of host restriction systems (32, 141); hence, it is possible that proteins of this family are indeed involved in a previously unknown early counterrestriction activity. The other such domain is defined by the ArdA protein, which has also been implicated in the countering of restriction by directly binding restriction enzymes (29, 67). It is found primarily in polyvalent proteins from Firmicutes and is closely associated with ArdC-N (Fig. 5H). In light of the above analysis indicating a role for ArdC-N in DNA binding, we propose that it might recruit the ArdA domain to the newly injected DNA to protect it from host restriction attack. The YodL domain is prototyped by Bacillus subtilis YodL and is widespread in Firmicutes polyvalent proteins from conjugative elements. Earlier studies have shown genetic interactions between YodL and the cytoskeletal protein MreB during cell elongation and division (142). Thus, in contrast to the above domains, the YodL domain might not be involved in counterrestriction strategies. Instead, it might play a role in anchoring of the invasive element or the polyvalent proteins to the cytoskeleton to facilitate their transport or localization (Fig. 5H).

### LPDs.

Beyond the above-described domains, we also found 40 further domains (LPD1 to LPD40) whose precise functions remain elusive, as most of them cannot be currently unified with known domains. Nevertheless, the conservation patterns and secondary structures provide tantalizing hints regarding some of them. For example, at least seven of these domains (LPD5, LPD15, LPD19, LPD21, LPD22, LPD34, and LPD39) are potentially enzymatic, as they show strongly conserved charged and polar residues typical of enzymes (see Table S1 and File S2 at ftp://ftp.ncbi.nih.gov/pub/aravind/polyvalent/polyvalent.html). In light of the above observations on the characterized domains in the polyvalent proteins, these potentially enzymatic domains might perform novel catalytic functions in some of the previously noted categories. Further contextual analysis shows that some of them are strongly associated with a limited number of domains indicating functional linkages between them: LPD10 and LPD24 with LPD11; LPD28 with an MPTase; LPD30 with LPD29, and LPD36, as noted above, with the adenine methylase (Fig. 2A and B). Several of them are also found outside the context of polyvalent proteins and often either in neighborhoods with phage structural or T4SS pathway genes, suggesting that they function even in their solo forms as potential facilitators of the early phases of invasion.

### Biological implications of the contextual network of polyvalent proteins.

Contextual analyses combining information from conserved gene neighborhoods and domain architectures reveal the underlying syntactical features of domain linkages (Fig. 2), throwing light on their biological roles.

### Structure of the polyvalent protein domain network.

To better understand the structure of the polyvalent protein network, we analyzed it for various network parameters that might throw light on its functional and evolutionary aspects. The 131 domains, which are the nodes of the network, show a degree distribution (i.e., connections to other nodes) typical of several other biological networks, namely, a power law-like distribution of number of edges (Kolmogorov-Smirnoff statistic for power law fit = 0.071; *P* = 1) (143). This means that there is a relatively small set of domains (network hubs) that are connected to a disproportionately large number of other domains (∼20 domains with >20 connections to other domains; e.g., MPTase, ArdC, ART, SNF ATPase, N6A methylase, DdrB-ParB, and MuF). Such degree distributions in networks have been previously explained (143) by a general model of network growth by adding of new nodes with preferential attachment of the new nodes to preexisting nodes that have a higher number of connections. Such a model is entirely consistent with what we observe for polyvalent proteins where there is an expansion via recruitment of new components to enhance competitiveness in biological conflicts with their preferential combination with the MPTase and ArdC-N domains in particular (see below for further discussion). A subset of these hub domains also have the highest network betweenness scores (see Table S3 at ftp://ftp.ncbi.nih.gov/pub/aravind/polyvalent/polyvalent.html), which means that the shortest path between any two nodes in the network passes through them (144). In social networks, nodes with higher betweenness scores have greater potential for control of communication through the network. By analogy, we posit that these hub domains play a central role in the coordination of the disparate biochemical activities in the polyvalent protein network.

We also examined other network parameters, such as hub and authority scores, which were network measures originally developed for the internet to rank highly connected and authoritative Web pages (see Table S3 at the URL mentioned above) (145). In the case of a protein network, they respectively measure (i) the extent to which a hub domain might connect to other domains in proteins that are widely linked to other hub domains (hub score) and (ii) the extent to which a node tends to be connected to multiple hubs (authority score). On the basis of these scores, we were able to make a subtle distinction of hubs. The MPTase, unsurprisingly, has the highest hub and authority scores. However, although ArdC-N is the second ranked hub in terms of degree distribution, it has lower hub and authority scores than other smaller hubs such as DdrB-ParB, SWI2/SNF2 ATPase, MuF-C, ART, and PBECR1. Thus, ArdC-N is both less connected to other nodes frequently linked to other hub domains and less frequently linked to other hubs. This relates to its peculiar preference for the N-terminal location in proteins (see below for further discussion).

Cliques are the most densely connected subnetworks within networks where every node is connected to every other node of the clique. We accordingly detected the largest cliques in our network (size of seven or eight nodes) and examined the subnetwork obtained by merging these cliques. This produced a subnetwork of 37 nodes (Fig. 2C) that, when arranged by the Kamada-Kawai and Fruchterman-Reingold algorithms (146, 147), defined two distinct densely connected subgraphs—one dominated by domains found primarily in conjugative elements (centered on ArdC) and the other in phages with the MPTase, SNF ATPase, and N6A methylase nodes being central to both subgraphs. Another measure of network connectivity is whether removal of a node causes the network to fall apart into unconnected subnetworks or whether it still remains intact (a biconnected network). We determined the largest biconnected subnetwork of network that remains intact even upon the removal of single nodes. This subnetwork included 96 nodes, including all hubs and thus encompasses ∼73% of the nodes in the complete network with distinctly clustering components from conjugative elements and phages. This indicates that the majority of the network nodes are held together by a multiplicity of linkages. Thus, it strongly supports the idea that the polyvalent proteins as defined by us form a natural and coherent architectural theme.

### Genomic contexts of polyvalent proteins.

Polyvalent proteins are found in invasive elements from both bacteria and archaea (see File S5 at ftp://ftp.ncbi.nih.gov/pub/aravind/polyvalent/polyvalent.html). However, they are more prevalent in the former, and currently, almost all examples of large proteins (>1,000 aa) come from bacteria. The most basic contextual linkages are related to the type of element deploying the polyvalent protein. (i) The association with flanking genes encoding RCR-related components such as relaxases and the Tra/Vir proteins that constitute the T4SS-like DNA pump and the rest of the DNA transfer apparatus (17, 42) is the hallmark of conjugative elements (Fig. 1C, 4, and 5). More detailed analysis distinguishes two types of such elements: primary conjugative plasmids and conjugative transposons prototyped by the Tn*1549* and (42, 43) and Streptococcus pneumoniae Tn*5253*-like elements. In these settings, the genes for polyvalent proteins are also often associated with those coding for TA systems and fertility inhibition factors (81). This suggests a higher-order linkage with other conflict systems that are related to either “addiction” of the element (TA systems) or sexual conflict—i.e., prevention of rival elements from utilizing the element's DNA transfer system. (ii) Phage polyvalent proteins are typically found in the vicinity of late genes, predominantly those coding for virion proteins, which is consistent with their inclusion in the virion. Moreover, as noted above for specific domains, they often show fusions to the virion-associated MuF domain in other phage proteins. Together, these features of the above two categories strongly support the idea of polyvalent proteins defining a common functional theme across selfish elements that combines a whole slew of disparate activities required alongside or just after invasion of a new host. (iii) While polyvalent proteins encoded in host genomes outside selfish elements make up a sizeable fraction of the polyvalent proteins, these have clearly been acquired from the invasive elements, as they are often closely related to versions in such elements. Hence, we propose that they have probably been acquired by the host genome from integrated elements and prophages. In support of this, we found numerous genome-integrated prophages with polyvalent proteins. One possible advantage to the host of capturing polyvalent proteins is that they could be used to target incoming parasitic elements. This is consistent with the idea that some of the domains encoded by polyvalent proteins not only target host functions but might also prevent superinfection by or help overcome antisuperinfection defenses of other parasitic elements.

### Shared and unique themes of polyvalent proteins.

In total, about 27% of the domains are shared by polyvalent proteins of the phage-type and conjugative elements, the most common being the zincin-like MPTase and the SWI2/SNF2 ATPase-DNA adenine methylase-LPD36 module, which are widely seen in both types of systems. Some of these might be rather common in one system but rare in the other; e.g., the ArdC-N domain is present in 93% of conjugative element polyvalent proteins but is rarely seen in the phage type. On the whole, the common themes point to certain similar challenges faced by either type of invasive element during and shortly after the invasion process. Both elements are subject to attack by R-M systems. Thus, the abundant presence of SWI2/SNF2 ATPase-DNA adenine methylase-LPD36 module across these elements suggests that counterrestriction strategies are central to the survival of both types of elements. The presence of an MPTase across both of these systems and its central position in terms of multiple measures (Fig. 2) point to an important feature of the logic of polyvalent proteins, i.e., that the primary challenge faced by elements is bringing together biochemically disparate activities to act nearly at once in the small temporal window following invasion. This is best achieved by having all activities assembled into a single protein or a few proteins delivered during invasion—i.e., the polyvalent proteins. However, this creates a new challenge—the domains in the polyvalent proteins have distinct subcellular targets that might not necessarily be proximal. Hence, the MPTase domain is the solution that allows the polyvalent protein to be processed into individual functional units.

The remaining domains are unique to either system. Such distinctions are seen even among the less frequent domains. The N4-like virion polymerase, RelA/SpoT, DarA-N, PBECR2, 2H, InPase, DdrB-ParB, LPD4, LPD38, and lysozyme domains are found only in phage-type polyvalent proteins. On the other hand, the DNAG primase, Primpol, YodL, LPD31, polβ-NTase, RadC, LPD25, LPD16, and LPD17 domains are found only in polyvalent proteins of conjugative elements, even though solo versions of some of these are often found in phages. These point to the unique challenges faced by each type of invasive element. The conjugative elements pump their genome as ssDNA through the transfer machinery into the recipient cell, where they need to revert to dsDNA immediately on entry (14). Thus, their polyvalent proteins are dominated by the ssDNA-binding ArdC-N domain and the two kinds of primases that can prime their DNA for RCR by the host apparatus (37, 95). In contrast, phages do not replicate their DNA immediately on entry; rather, their primary challenge is to establish transcription of their genome. This constraint expresses itself in polyvalent proteins in the form of the N4-like RNAP and protein-modifying enzymes that hijack the host proteins via covalent modifications (54, 60). The unique presence of PBECR RNases in phage polyvalent proteins suggests that shutting down host transcription or blocking the CRISPR/Cas-like systems through targeted RNA degradation is of greater importance for phages than for conjugative plasmids, which are mostly in symbiosis with the host genome.

### The provenance and evolution of polyvalent proteins.

Like other prokaryotic conflict systems, such as R-M and TA systems (12, 148, 149), polyvalent proteins are also shared between the selfish elements and the host genome. The “capture” of polyvalent proteins by the host genomes suggests that these systems are amenable to use in potentially defensive contexts on both sides of the biological conflict. However, in this case, their ultimate provenance can be clearly placed in the selfish elements because of the presence of a large number LPDs that could not be unified with domains from any other system found outside these elements. Some of the domains found in polyvalent proteins (e.g., protein-modifying enzymes, SWI2/SNF2 ATPases, ParB superfamily enzymes, and DNA methylases) are shared with a wide range of conflict systems, suggesting that these enzymatic domains are evolutionarily successful strategies, irrespective of the actual nature of the conflict. Large proteins linking multiple domains with disparate functions have evolved in several prokaryotic conflict systems such as polymorphic toxins, secondary metabolite biosynthesis (including antibiotic), and certain unusual restriction systems (3, 10, 44), yet polyvalent proteins share very few domains with them. A large fraction of the domains are unique to the context of polyvalent proteins and are thematically different from other large multidomain conflict-related proteins in domain architectures. Thus, polyvalent proteins emerged primarily as a unique adaptation of selfish elements for biological conflicts associated with the early phase of invasion, which are distinct from the challenges encountered in other conflicts.

Several domains in polyvalent proteins are also found either as solo versions or in architectures with fewer linked domains in related mobile elements. This indicates that they have been pieced together from these solo versions under the consistent selective pressure for coeval action during and shortly after invasion, as argued above. Evidence of strong selective pressure for domain accretion is presented by the observation that, in several instances, the same domain appears to have been incorporated into polyvalent proteins independently on several occasions in different types of invasive elements (e.g., MPTase, SWI2/SNF2 ATPase, DNA methylase, GNAT, primases, RelA/SpoT modules). Repeated accretion from solo modules or smaller multidomain proteins also points to an arms race with the host that has selected for multipronged strategies to be deployed at once along with the transferred DNA. This potentially allows the invading elements to simultaneously present multiple alternative options against host defenses that might be directed at particular strategies of the invasive element. This is consistent with evidence of evolution of multipronged strategies on the host side that target different aspects of the element's biology. An analogy may be drawn between the polyvalent proteins described in this work and the polyproteins of eukaryotic RNA viruses (150). Both cases represent an evolutionary solution to similar challenges of combining biochemically disparate domains that need to function together temporally. Indeed, in both cases, similar mechanistic solutions in the form of release of the combined domains by the activity of embedded peptidase domains are also seen (150, 151). However, beyond this operational analogy, the two systems have few, if any, features in common in terms of the actual domains incorporated into the polyproteins. Notably, they even differ in terms of the types of peptidases that are used.

Across prokaryotes, polyvalent proteins are widely seen in many diverse lineages, although they are overrepresented in Firmicutes, Proteobacteria, Bacteroidetes, Spirochaetes, and Fusobacteria (see File S7 at ftp://ftp.ncbi.nih.gov/pub/aravind/polyvalent/polyvalent.html). These lineages also show greater complexity in their architectures than other prokaryotic lineages (see File S7 at the URL mentioned above). Of the lineages with many representative genomes, the archaea and cyanobacteria show a particular paucity of polyvalent proteins. One possibility is that in these lineages, the primary mobile elements and phages infecting these hosts are likely to be distinct from those that deploy polyvalent proteins. In particular, phages deploying polyvalent proteins belong to the terminase portal class. These phages lack a membrane internal to the capsid typical of the viruses with the HerA/FtsK class of DNA pumps. This feature might expose their DNA to a more immediate attack by host systems. Finally, we found that at least one domain, the ArdC-N domain, which is specific to polyvalent proteins, has spread beyond the circle of prokaryotic mobile elements and their host genomes. This domain was acquired by eukaryotes on two independent occasions, once in the form of the DNA-binding domains of the XPC/Rad4 protein and once in the form of the DNA-binding domains of the Trypanosoma Tc38 family of proteins. In the first case, the transfer appears to have preceded the last eukaryotic common ancestor and the domain was incorporated into a protein that is part of the DNA repair network unrelated to the original role of the ArdC-N domain (Burroughs et al., unpublished). This suggests that they were recruited primarily for their distinctive ssDNA recognition capability, which proved useful in the context of DNA mismatch recognition (134, 136–138). Interestingly, this is a striking parallel to the case of another ssDNA-annealing domain, the Rad52 domain, which we had earlier shown to have been acquired by the eukaryotic DNA recombination/repair system from a bacteriophage source (152). In the second case, the Tc38 family, the transfer appears to have taken place within the kinetoplastid lineage of euglenozoans, where they were incorporated into the replication system for the plasmid-like mitochondrial (kinetoplast) DNA circles, known as minicircles, unique to kinetoplastids (135). Here, the eukaryotic adaptation hews more closely to the ancestral function of binding plasmid ssDNA in the context of posttransfer replication. More generally, these findings add yet another example to the growing body of evidence that several seemingly unique eukaryotic systems have evolved by wholescale “reuse” of components acquired from prokaryotic conflict systems (9, 10, 46, 58, 75).

### Conclusions.

We identify a class of proteins with multidomain architectures from diverse prokaryotic invasive elements and present evidence that they represent a novel paradigm in the deployment of such proteins in biological conflict. While the linkage of multiple domains into a single polypeptide occurs across several previously studied conflict systems, polyvalent proteins are unique in their architectural themes. They link a set of domains with disparate activities into the same polypeptide to enable nearly simultaneous execution of multiple actions relating to both the targeting of host machineries and defense systems by distinct effectors and the facilitation of replication or transcription of the invasive element. As a consequence, they appear to be proteins that are delivered along with the DNA of the invasive element via either the conjugation apparatus of the conjugative elements or injection via the phage tail. While these activities are synthesized or delivered as a single polypeptide, we find evidence that they are separated during actual deployment on the basis of the pervasive presence of MPTases in the polyvalent proteins. These features strongly suggest that they play a key early role in the establishment of the infection of the invasive element, be it a phage or a plasmid. Thus, these findings provide an avenue by which to further explore the poorly understood aspects of biological conflicts during the early stages of establishment of an invasive element in the host cell.

Several conjugative transposons that encode polyvalent proteins are also transmitters of antibiotic resistance between bacteria. Hence, understanding the role of polyvalent proteins might provide insights into the dynamics of their spread. Finally, components of such conflict systems have been a rich source of reagents for molecular biology, such as restriction enzymes, the CRISPR/Cas components, and nucleic acid polymerases (153–156). We suggest that components of these polyvalent proteins might have similar utility. Of particular interest in this regard might be the predicted PBECR RNases, which might target CRISPR/Cas or other RNAs. Moreover, the domain architectural theme of combining multiple activities, followed by separation by means of inbuilt peptidase domains, might also help in engineering comparable multidomain proteins that might deliver disparate functional moieties at the same time.

## MATERIALS AND METHODS

Iterative sequence profile searches were performed with the PSI-BLAST and JACKHMMER programs run against the nr protein database of the National Center for Biotechnology Information (NCBI) (157, 158). Similarity-based clustering for both classification and culling of nearly identical sequences was performed with the BLASTCLUST program (ftp://ftp.ncbi.nih.gov/blast/documents/blastclust.html). The length (L) and score (S) threshold parameters were variably adjusted, depending on need. For example, the threshold parameters for clustering of nearly identical proteins were L = 0.9 and S = 1.2. The HHpred program was used for profile-profile searches (159). Structure similarity searches were performed with the DaliLite program (160, 161). Multiple-sequence alignments were built by the Kalign (162) and PCMA (163) programs, followed by manual adjustments on the basis of profile-profile and structural alignments. Secondary structures were predicted with the JPred program (164). For previously known domains, the Pfam database (165) was used as a guide, though the profiles were augmented by the addition of newly detected divergent members that were not detected by the original Pfam models. Clustering with BLASTCLUST, followed by multiple-sequence alignment and further sequence profile searches, was used to identify other domains that were not present in the Pfam database. For these alignments, see File S2 at ftp://ftp.ncbi.nih.gov/pub/aravind/polyvalent/polyvalent.html. Contextual information from prokaryotic gene neighborhoods was retrieved by a custom Perl script that extracts the upstream and downstream genes of the query gene and uses BLASTCLUST to cluster the proteins to identify conserved gene neighborhoods. Phylogenetic analysis was conducted by using an approximately maximum-likelihood method implemented in the FastTree 2.1 program under default parameters (166). Structural visualization and manipulations were performed with the PyMol (http://www.pymol.org) program. The in-house TASS package, which comprises a collection of Perl scripts, was used to automate aspects of large-scale analysis of sequences, structures, and genome context. Network analysis was performed in the R language with the igraph and circlize packages (167, 168).
